# Longevity and Composition of Cellular Immune Responses Following Experimental *Plasmodium falciparum* Malaria Infection in Humans

**DOI:** 10.1371/journal.ppat.1002389

**Published:** 2011-12-01

**Authors:** Anne C. Teirlinck, Matthew B. B. McCall, Meta Roestenberg, Anja Scholzen, Rob Woestenenk, Quirijn de Mast, Andre J. A. M. van der Ven, Cornelus C. Hermsen, Adrian J. F. Luty, Robert W. Sauerwein

**Affiliations:** 1 Department of Medical Microbiology, Radboud University Nijmegen Medical Centre, Nijmegen, The Netherlands; 2 Department of Laboratory Medicine, Laboratory of Hematology, Radboud University Nijmegen Medical Centre, Nijmegen, The Netherlands; 3 Department of General Internal Medicine, Radboud University Nijmegen Medical Centre, Nijmegen, The Netherlands; University of Notre Dame, United States of America

## Abstract

Cellular responses to *Plasmodium falciparum* parasites, in particular interferon-gamma (IFNγ) production, play an important role in anti-malarial immunity. However, clinical immunity to malaria develops slowly amongst naturally exposed populations, the dynamics of cellular responses in relation to exposure are difficult to study and data about the persistence of such responses are controversial. Here we assess the longevity and composition of cellular immune responses following experimental malaria infection in human volunteers. We conducted a longitudinal study of cellular immunological responses to sporozoites (*Pf*Spz) and asexual blood-stage (*Pf*RBC) malaria parasites in naïve human volunteers undergoing single (n = 5) or multiple (n = 10) experimental *P. falciparum* infections under highly controlled conditions. IFNγ and interleukin-2 (IL-2) responses following *in vitro* re-stimulation were measured by flow-cytometry prior to, during and more than one year post infection. We show that cellular responses to both *Pf*Spz and *Pf*RBC are induced and remain almost undiminished up to 14 months after even a single malaria episode. Remarkably, not only ‘adaptive’ but also ‘innate’ lymphocyte subsets contribute to the increased IFNγ response, including αβT cells, γδT cells and NK cells. Furthermore, results from depletion and autologous recombination experiments of lymphocyte subsets suggest that immunological memory for *Pf*RBC is carried within both the αβT cells and γδT compartments. Indeed, the majority of cytokine producing T lymphocytes express an CD45RO^+^ CD62L^-^ effector memory (EM) phenotype both early and late post infection. Finally, we demonstrate that malaria infection induces and maintains polyfunctional (IFNγ^+^IL-2^+^) EM responses against both *Pf*RBC and *Pf*Spz, previously found to be associated with protection. These data demonstrate that cellular responses can be readily induced and are long-lived following infection with *P. falciparum*, with a persisting contribution by not only adaptive but also (semi-)innate lymphocyte subsets. The implications hereof are positive for malaria vaccine development, but focus attention on those factors potentially inhibiting such responses in the field.

## Author Summary

A decade into the 21st century, malaria remains responsible for an intolerable global health burden and an effective vaccine is sorely needed. Compounding the many technical hurdles in developing such a vaccine, (naturally-acquired) immunity to malaria is generally perceived to be short-lived, although direct evidence from field studies is conflicting. To overcome this issue, we measured the development of immune responses against the malaria parasite *Plasmodium falciparum* in human volunteers undergoing experimental malaria infections for the first time, allowing a uniquely detailed analysis thereof. We found that cellular immune responses against two clinically-relevant life-stages of the parasite are not only rapidly acquired following even a single malaria infection, but also remain virtually undiminished over a year later – an unprecedented measurement. These findings refute conclusively the notion that an intrinsic defect exists in either the development or persistence of cellular immune responses against malaria. This realization, in conjunction with a growing recognition that such responses are indeed associated with clinical protection against malaria, markedly enhances the prospect of one day developing a successful vaccine. Simultaneously, however, these results re-focus attention on the question of why the development of long-lived immune responses is often inhibited under conditions of natural exposure.

## Introduction

Malaria is caused by parasites of the genus *Plasmodium* that are transmitted from one human host to the next by *Anopheline* mosquitoes, putting an estimated 3.3 billion of the world's population at risk [1]. Upon inoculation by a mosquito, sporozoites initiate an asymptomatic infection of hepatocytes from which blood-stage forms emerge to invade and multiply exponentially within erythrocytes. The latter process underlies the full spectrum of morbidity and mortality associated with clinical malaria. Compounding this global public health burden is the fact that first infections do not immediately induce immunity. Instead, infants in endemic areas remain susceptible to multiple new symptomatic infections throughout childhood and early adulthood, and adults frequently still harbor sub-clinical parasitemia (reviewed in [2], [3]). Both poor induction (priming) of immune responses by the parasite and rapid attrition of such responses have been proposed as explanations, although the validity of both hypotheses has been brought into question (discussed in [4], [5], [6]).

Direct immunological evidence from studies in humans that support or reject these theories is limited. The commonly held view that immune responses to *Plasmodium* parasites are short-lived following exposure, is mainly based on the short half-life of specific antibodies (reviewed in [7]). It would appear that cellular responses to individual antigens are also either relatively short-lived, i.e. declining within a few years of exposure [8], [9], [10], or at least unstable [11], [12], [13], [14], [15], but may persist occasionally [16]. Many field studies, however, suffer from a profound difficulty in controlling for exposure amongst study subjects, limiting interpretation thereof. Anecdotal evidence from historical malaria-therapy studies suggests that cellular proliferative responses to crude whole parasite antigen can be detected in donors several years after a single infection [17]. More recently, robust cellular cytokine responses were detected three months post infection in previously naïve volunteers [18]. Within these cellular immune responses, interferon-gamma (IFNγ) in particular is considered to play a major role (reviewed in [19]).

Experimental human malaria infections by bites of *P. falciparum* infected mosquitoes offer a controlled measure of exposure and a safe and well-established model, and have been performed on hundreds of volunteers over the past two decades primarily for assessing the efficacy of candidate malaria vaccines [20]. This model allows controlled studies on the development and maturation of intrinsic immune responses in the course of a malaria infection, and on how (long) cellular memory is maintained. Here we conducted a comprehensive longitudinal study of cellular responses, focusing on IFNγ production by multiple subsets of innate and adaptive immune cells, induced by both *P. falciparum* sporozoites (*Pf*Spz) and asexual blood-stage parasites (*P. falciparum*-infected red blood cells; *Pf*RBC) in malaria-naïve volunteers undergoing single or multiple experimental infections with *P. falciparum*.

We show that even a single patent malaria episode induces robust cellular re-call responses to both parasite stages, persisting at almost undiminished levels at least 14 months post infection and involving both adaptive and innate compartments.

## Results

### Cellular IFNγ re-call responses to both sporozoites and blood-stage parasites are readily induced and long-lived following infection

**Figure 1:**
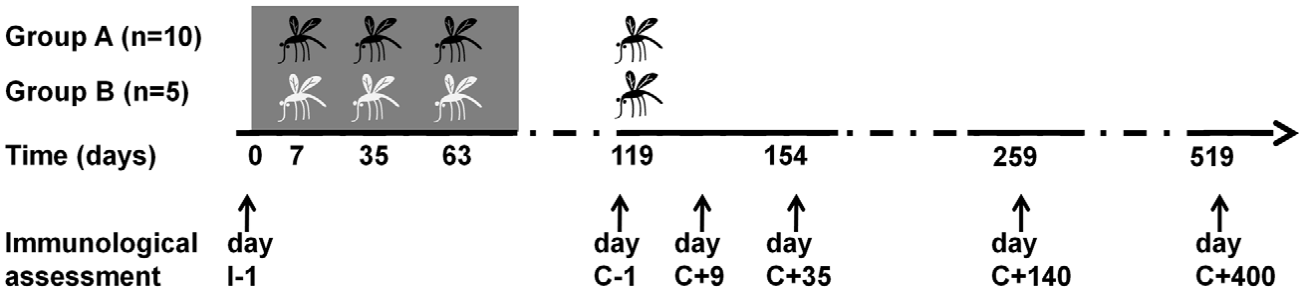
Flowchart of Experimental Human Malaria Infection study. Black and white mosquito symbols indicate exposure to infected mosquito bites and uninfected mosquito bites, respectively. Development of patent blood-stage parasitemia following the first three inoculations was prevented by prophylactic chloroquine treatment, indicated by grey shading. Arrow heads indicate time points of immunological assessment: prior to immunization (I-1), prior to patent challenge (C-1), during expected blood-stage infection (C+9), two weeks after treatment (day C+35), 4.5 months post-challenge (day C+140) and again 1.1 year post-challenge (day C+400).

**Figure 2:**
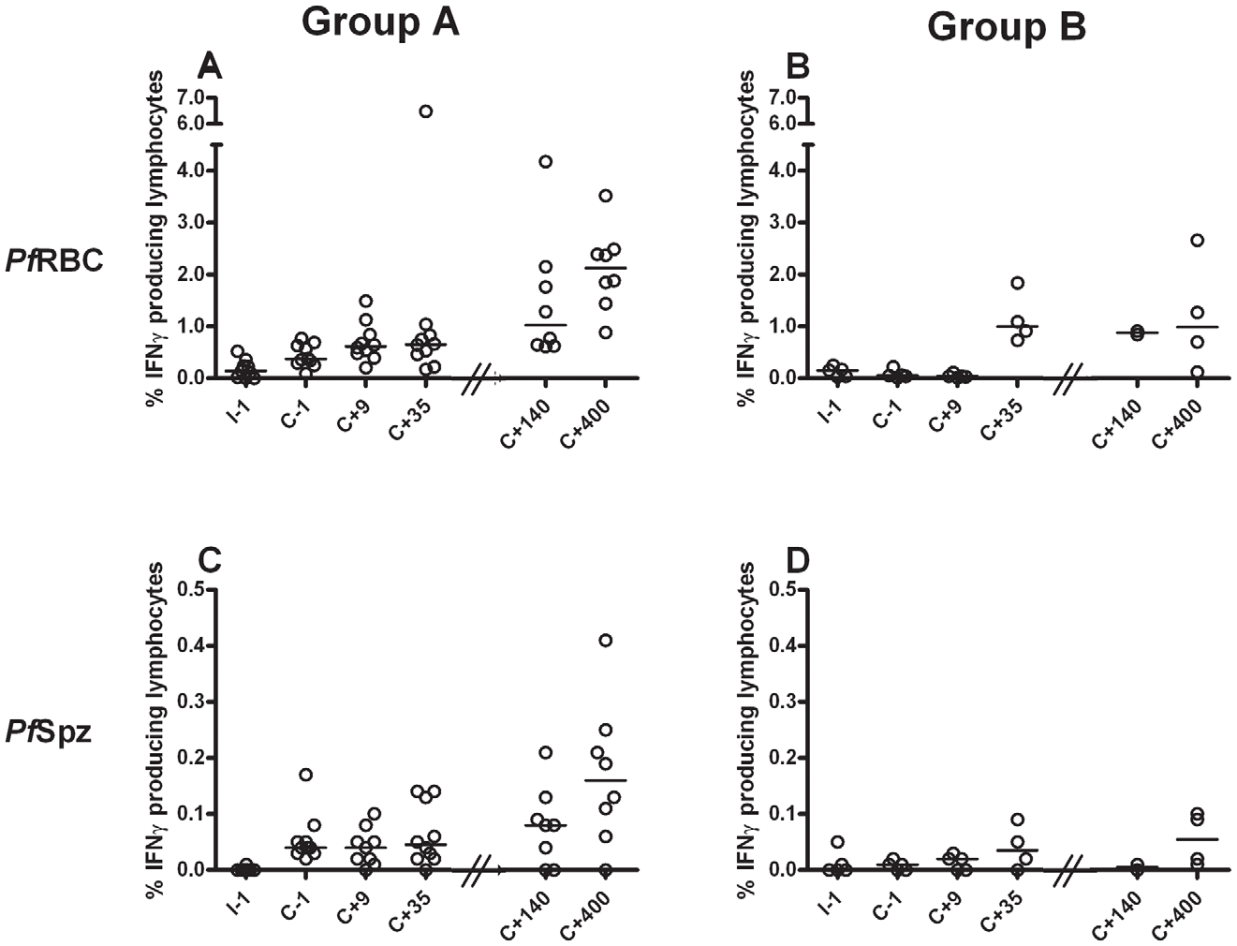
Induction and persistence of IFNγ responses to *Pf*RBC and *Pf*Spz during experimental malaria infection. PBMC were isolated from volunteers prior to inclusion (day I-1), immediately prior to patent challenge (day C-1), during expected blood-stage malaria infection (day C+9), two weeks after treatment (day C+35), 4.5 months post-challenge (day C+140) and again 1.1 year post-challenge (day C+400). Note that Group A, but not Group B volunteers were exposed thrice to immunizing sub-patent infections between day I-1 and C-1 (**Figure 1**). PBMC of volunteers of Group A (**A+C**) and Group B (**B+D**) were stimulated *in vitro* for 24 hours with *Pf*RBC (**A+B**) or *Pf*Spz (**C+D**) or their respective uninfected red blood cells (uRBC) or salivary glands from uninfected mosquitoes (MSG) controls, then stained for intracellular IFNγ and analyzed by flow cytometry. Shown are the percentage of total lymphocytes staining positive for IFNγ at each time point. Background responses were subtracted from the responses to parasite stimuli for every individual volunteer at every individual time point. Symbols represents responses by individual Group A volunteers (n = 10) and Group B volunteers (n = 5) for whom sufficient cells were available. Horizontal lines represent group medians. Median background values for uRBC were 0.01% [0.01–0.03] (median [IQR]) on I-1 up to C+35 and 0.03% [0.01–0.16] on C+140 and C+400. Background values for MSG were 0.02% [0.01–0.02] on I-1 up to C+35 and 0.07% [0.03–0.25] on C+140 and C+400.

*In vitro* parasite-specific responses were measured in peripheral blood mononuclear cells (PBMC) isolated from two sets of human volunteers prior to and at several time points after exposure to *P. falciparum* infection. Group A volunteers (n = 10) were exposed thrice to immunizing bites (I) of infected mosquitoes whilst under chloroquine prophylaxis and thereafter challenged (C) once again; Group B volunteers (n = 5) received only a single infection in parallel with Group A challenge (**Figure 1**). Total lymphocyte responses to *Pf*Spz and *Pf*RBC were barely detectable above background prior to exposure (day I-1) in both groups of volunteers (**Figure 2**). Re-call responses by lymphocytes to both *Pf*Spz and *Pf*RBC, as measured by IFNγ production following overnight re-stimulation, were detectable in Group A volunteers following exposure to immunizing bites (day C-1 compared to I-1, one-way ANOVA with Dunnet's post-test, p<0.05 for *Pf*Spz and p<0.01 for *Pf*RBC) and remained high after re-challenge until day C+35 (p<0.001 and p<0.01, respectively) (**Figure 2** **.A+C**). Of note, one volunteer displayed a disproportionally amplified IFNγ response to *Pf*RBC at time point C+35. For this reason, this volunteer was left out of statistical analysis as an extreme outlier. Re-call responses to *Pf*RBC (p<0.001, I-1 compared to C+35), and to a lesser extent also to *Pf*Spz, became detectable in Group B volunteers following their first infection (**Figure 2.B+D**). This shows that cellular immune responses to whole parasites are readily inducible in previously-naïve human volunteers, following a small number of, or even a single *P. falciparum* infection. Most remarkably, in further experiments with samples collected at later time points (days C+140 and C+400), we found that parasite-specific cellular responses did not wane after exposure. Instead, they remained robust more than a year post-challenge, albeit with considerable inter-individual variation (**Figure 2**).

Cellular responses to protein pools of either sporozoite-stage (CSP and TRAP), liver-stage (LSA-1 or Exp-1) or blood-stage (AMA-1, MSP-2, MSP-3 and GLURP) antigens (all leading malaria vaccine candidates), however, were never detectable above background.

### αβT and γδT cells are the main in vitro IFNγ-producers in response to *Pf*RBC following infection

Many different lymphocyte subsets, including αβT cells, γδT cells and NK cells, have variously been shown capable of responding to *Pf*RBC. Therefore, we assessed IFNγ responses by those cell types to *Pf*RBC prior to (I-1 for Group A, I-1 and C-1 for Group B) and post exposure (C-1 and later for Group A, C+9 and later for Group B; flow cytometry gating strategy illustrated in **Figure S1**). Relative proportions of lymphocyte subsets within the total peripheral population did not differ markedly over time at the various time points assessed (**Table S1**). The only exception were γδT cells, the relative numbers of which increased within the peripheral lymphocyte population post exposure in both sets of volunteers (p = 0.0013 for Group A; p = 0.029 for Group B, one-way ANOVA, I-1 to C+35). Response patterns in most lymphocyte subsets, including αβT cells, NKT cells and NK cells, mirrored the dynamics of the total lymphocyte response in relation to exposure: whereas almost no responses above background could be detected in volunteers at inclusion, IFNγ responses to *Pf*RBC became clearly detectable following challenge (**Figure S2**). In contrast, a large proportion of γδT cells (median 7.9% and 6.8% for Group A and B, respectively) demonstrated the capacity to respond to *Pf*RBC even prior to exposure. Following infection, this percentage increased still further (p = 0.013 Group A; p = 0.003 Group B, one-way ANOVA I-1 to C+35). Responses in ‘γδNKT’ cells, relatively infrequent in total number, resembled this pattern of regular γδT cells (**Table S1** and **Figure S2**).

**Figure 3:**
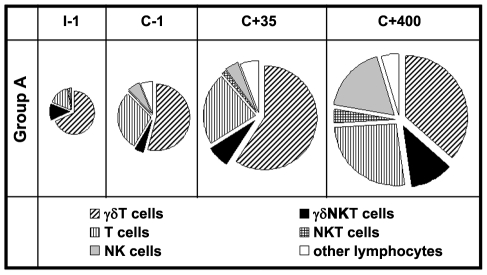
Contribution of innate, semi-innate and adaptive lymphocyte subsets to the total IFNγ^+^ response to *Pf*RBC. PBMC isolated from Group A volunteers at the respective study time points were stimulated with *Pf*RBC or uRBC and stained for intracellular IFNγ and surface expression of CD3, γδT and CD56 (gating strategy shown in **Figure S1.A**). Pie charts show the relative contributions of αβT cells (CD3^+^γδ^-^CD56^-^), γδT cells (CD3^+^γδ^+^CD56^-^), NK cells (CD3^-^γδ^-^CD56^+^), NKT cells (CD3^+^γδ^-^CD56^+^), ‘γδNKT’ cells (CD3^+^γδ^+^CD56^+^) and other lymphocytes to the total number of IFNγ^+^ cells responding to *Pf*RBC (corrected for uRBC background). Shown are median values for ten Group A volunteers; pie chart surface areas directly correlate with the magnitude of (total) IFNγ+ responses. At time point I-1 the median [IQR] contribution of γδT cells, γδNKT cells, αβT cells & NK cells to total IFNγ responses was 63% [45–74], 11% [6.4–15], 15% [5.1–37] & 1.9% [0.3–5.6], respectively; at C+35 57%[ 47–59], 6.7% [4.0–8.9], 22% [17–28] and 4.1% [3.0–7.1] and C+400 35% [29–44], 11% [8.0–16], 25% [20–28] & 17% [12–25].

Next, we assessed the relative contribution of the different lymphocyte subsets to the total IFNγ response at various time points during the study (**Figure 3**) in volunteers of Group A. Few lymphocytes produced IFNγ in response to *Pf*RBC prior to exposure (I-1), of which 63% (median) were γδT cells and 15% αβT cells, with γδNKT cells (11%) and NK cells (1.9%) making up most of the remainder. Interestingly, despite an increase in the overall proportion of responding cells over time, the relative contributions of the various lymphocyte subsets remained more or less stable following repeated exposure (C+35) (57%, 22%, 6.7% and 4.1%, respectively). By day 400 post-challenge, the dominating cell subsets contributing to overall IFNγ production remained αβT and γδT cells (35%, 25%, 11% and 17%, respectively). The contribution of the various cell subsets to responses in Group B volunteers also remained comparable over time (data not shown). Thus, not only ‘adaptive’ αβT cells and ‘semi-innate’ γδT cells, but clearly also ‘innate’ NK and NKT cells contributed to the overall increase in lymphocytes responding to *P. falciparum* by IFNγ production following exposure (**Figure 3**).

A more in depth phenotypic analysis of responding T cell subsets in donors with sufficient responses at the latest time point (C+400, **Figure S3**) revealed that IFNγ-producing CD4^+^ T cells markedly outnumbered CD8^+^ T cells in response to both sporozoite and blood-stage parasites post-challenge. Following *in vitro* re-stimulation with *Pf*RBC, 16% [13–22] (median [IQR]) and 26% [20–32] of IFNγ-producing T cells were of the CD4^+^CD8^-^ T-helper phenotype in Group A and B volunteers, respectively. In contrast, only 4.5% [3.1–5.6] and 7.3% [4.7–8.8] were CD4^-^CD8^+^ cytotoxic T lymphocytes (CTLs). The majority of IFNγ-producing T cells in response to *Pf*RBC, however, were CD4^-^CD8^-^ cells. Analysis in a subset of donors showed that these cells were predominantly γδT cells (data not shown). The contribution of CD4+ T cells was even more pronounced for *Pf*Spz-induced responses, with 70% [65–75] of IFNγ^+^ T cells belonging to the CD4^+^CD8^-^ population in Group A volunteers, and only 1.7% [0.9–2.5] to the CD4^-^CD8^+^ population (day C+400, **Figure S3**). Thus, whereas both CD4^-^CD8^-^ T cells and CD4^+^ T cells dominated responses to *Pf*RBC, the IFNγ response to *Pf*Spz was primarily mediated by CD4^+^ T cells only.

### Cells showing *in vitro* parasite-specific IFNγ re-call responses predominantly display an effector memory phenotype both early and late after infection

**Figure 4:**
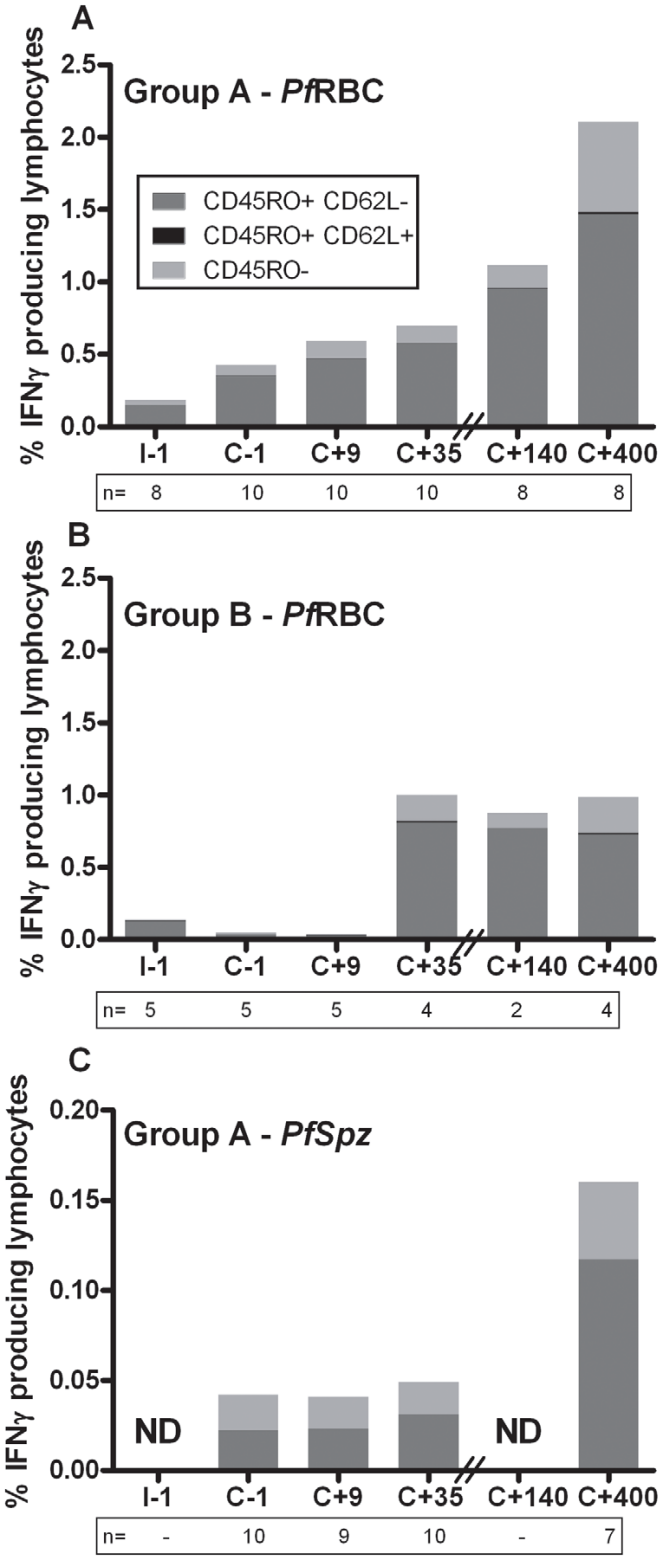
Contribution of EM and CM cells to the total IFNγ response to *Pf*RBC and *Pf*Spz. PBMC isolated from volunteers at various study time points were stimulated *in vitro* for 24 hours with *Pf*RBC (**A+B**) or *Pf*Spz (**C**) and stained for the memory marker CD45RO and the homing marker CD62L. Bars show the contributions of effector memory (EM, CD45RO^+^CD62L^-^), central memory (CM, CD45RO^+^CD62L^+^) and naive lymphocytes (CD45RO^-^) to the total percentage of IFNγ-producing cells over time. Height of bars represents median values for Group A (**A+C**) and Group B (**B**) volunteers for the different cell subsets. Donors with insufficient numbers of IFNγ responding cells to assess the relative contribution of cell subsets were excluded from this composition analysis. Numbers below the bars represent the number of donors included per time point. **ND** – not done: insufficient numbers to reliably assess group medians; this was similarly the case for anti-*Pf*Spz responses in group B. Since only donors with sufficient numbers of responding cells to assess the relative contribution of lymphocyte subsets are represented, these distributions may appear biased towards patterns in relatively stronger responders.

Early after treatment (day C+35) in Group A volunteers, 84% [80–87] (median [IQR]) and 0.1% [0.0–0.4] of IFNγ-producing lymphocytes displayed effector memory (EM, CD45RO^+^CD62L^-^) and central memory (CM, CD45RO^+^CD62L^+^) phenotypes, respectively, following 24-hour *in vitro Pf*RBC re-stimulation. Remarkably, despite an overall increase in the response to *Pf*RBC in Group A volunteers on day C+400, the relative contributions of CD62L^-^ EM and CD62L^+^ CM cells remained largely stable: 72% [67–75] and 0.6% [0.6–0.8], respectively (**Figure 4** **.A**). Corresponding values for Group B volunteers at day C+35 were 76% [74–79] and 0.5% [0.3–1.1] and remained constant over time, both in terms of percentage of responding cells and in EM/CM distribution (**Figure 4.B**). Responses to *Pf*Spz stimulation showed an EM/CM pattern very similar to *Pf*RBC responses as determined for group A volunteers (**Figure 4.C**). γδT cells also displayed an EM phenotype (CD45RO^+^CD62L^-^ or CD62L^intermediate^) as shown in **Figure S1.C**. Thus, *in vitro* parasite-specific re-call responses were primarily found in EM-type populations, which include both αβT cells and γδT cells, even months after infection. Cells of CD62L^+^ CM phenotype, in contrast, were detectable in only a negligible fraction of the total re-call response at all time points examined.

### Immunological memory for *Pf*RBC appears to be carried within both the αβT cell and γδT cell compartments

**Figure 5:**
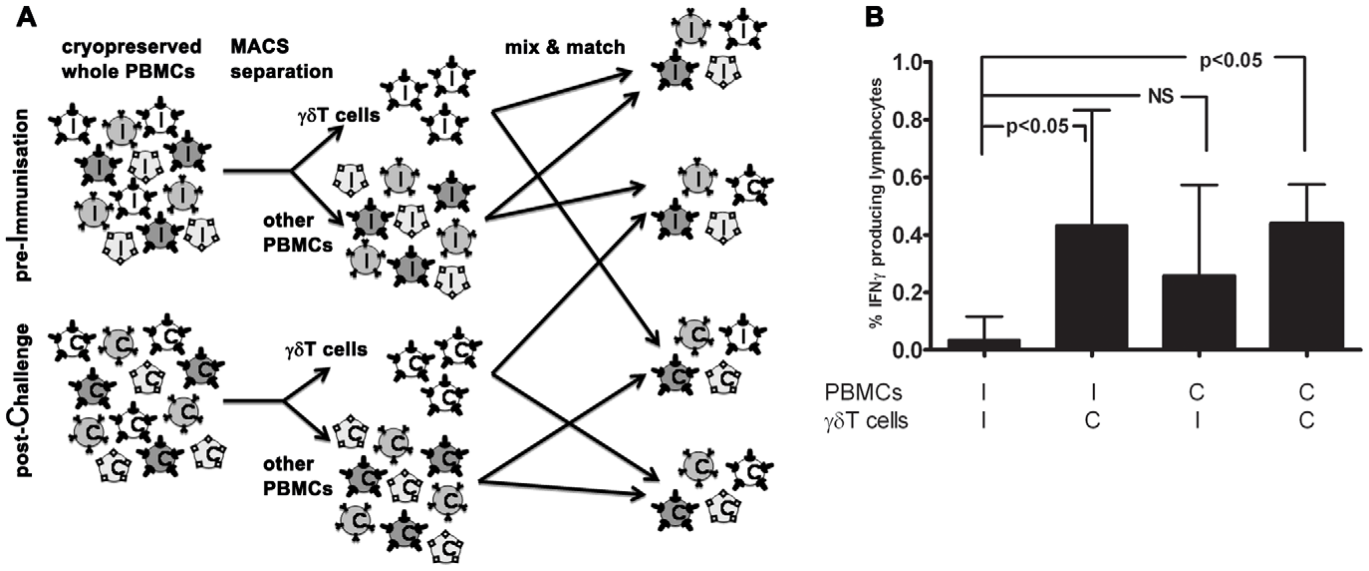
Immunological memory carriage by the γδT compartment vs other PBMC. (**A**) Cryopreserved PBMC isolated from volunteers at inclusion (I) or 35 or 140 days post-challenge (C), were thawed and separated by magnetic beads into γδT^+^ lymphocytes (white) and remaining γδT^-^ PBMC (shades of grey, e.g. αβT cells, NK cells, B cells, monocytes). Following autologous re-combination at original ratios, PBMC were stimulated, stained and measured as for **Figure 3**. (**B**) Shown are percentages of total lymphocytes staining IFNγ^+^ following incubation with *Pf*RBC (corrected for uRBC background). Data represent median+IQR of seven volunteers from whom sufficient cells were available for the assay.

Since both αβT cells and γδT cells display memory phenotypes and can mount adaptive responses, we assessed their respective ability to initiate cellular re-call responses to *Pf*RBC. To this end, we separated and re-combined γδT cells and other PBMC (consisting of approximately 80% αβT cells and 5% NK cells) from both inclusion (I; ‘*Pf*-naïve’) and 35 or 140 days post-challenge (C; ‘*Pf*-experienced’) of volunteers from both groups for whom sufficient cells were available (**Figure 5** **.A**). Following *in vitro* stimulation, total numbers of IFNγ^+^ lymphocytes in naïve PBMC populations supplemented with *Pf*-experienced γδT cells were significantly higher than in populations containing only *Pf-*naïve cells (I-I versus I-C; p<0.05, One-way ANOVA). This suggests that the γδT compartment carries some immunological memory for *Pf*RBC (**Figure 5.B**). Indeed, the *Pf*RBC response by *Pf*-experienced γδT cells in some donors was more than twice as high compared to that by *Pf*-naïve γδT cells, even in the presence of otherwise naïve PBMC populations (data not shown). IFNγ responses in PBMC populations containing *Pf*-experienced γδT-depleted cells (mainly αβT cells) also appeared higher than in populations containing only *Pf-*naïve cells (I-I versus C-I; not significant).

### Long-lived polyfunctional memory re-call responses to malaria parasites are more prominent in anti-*Pf*Spz compared to anti-*Pf*RBC responses

Whereas IFNγ has many direct effector functions, IL-2 is important for T cell proliferation and induction of cellular memory responses. In a final set of experiments, we therefore explored the dynamics of EM lymphocytes producing either IL-2 or IFNγ alone (unifunctional), or both cytokines simultaneously (polyfunctional cells), in response to *Pf*RBC and *Pf*Spz. In Group A volunteers, the percentage of total IL-2^+^ EM cells responding to *Pf*RBC, although low in absolute numbers, increased significantly from 0.08% [0.04–0.12] (median [IQR]) of EM cells at day I-1, to 0.31% [0.17–0.45] at day C-1 (p<0.001, one-way ANOVA with Dunnett's post-test) and 0.22% [0.19–0.42] at day C+35 (p<0.01, **Figure S4.A**) and remained clearly detectable at day C+140 and day C+400. This was in line with the increase in total lymphocyte IFNγ responses to *Pf*RBC after immunization (**Figure 2**). Similarly to both total IFNγ and total IL-2 responses, the percentage of EM-type cells producing both IFNγ and IL-2 in response to *Pf*RBC increased from 0.025% [0.003–0.078] on day I-1 to 0.14% [0.09–0.22] on C-1 (p<0.01) and 0.13% [0.10–0.18] on day C+35 (p<0.01, **Figure S4.B**) and remained present up to day C+400. The relative contribution of such polyfunctional cells to the overall number of cytokine producing EM cells, however, remained relatively stable with an apparent slight, but non-significant increase on day C-1 and C+9 (**Figure S5**).

**Figure 6:**
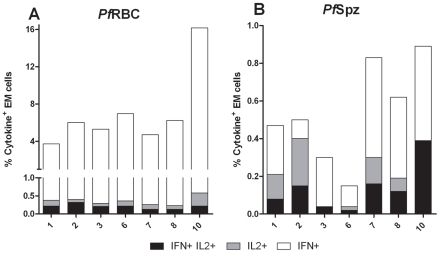
Uni- and polyfunctional EM T cell responses to *Pf*RBC and *Pf*Spz one year post-infection. Data represent percentage of effector memory (EM) cells producing either IFNγ alone, IFNγ and IL-2, or IL-2 alone, following 24 hours *in vitro* stimulation with (**A**) *Pf*RBC or (**B**) *Pf*Spz at 400 days after challenge (C+400) for seven individual volunteers of Group A.

Total IL-2 and polyfunctional responses to *Pf*Spz by EM cells from Group A volunteers remained low up to C+35 (p = 0.8 and 0.1, respectively, compared to I-1). IL-2 increased from C+140 to C+400 (p = 0.039, paired Student's *t*-test; **Figure S4.A**). A similar trend was seen for polyfunctional responses (p = 0.15; **Figure S4.B**). Interestingly, months after malaria infection, the contribution of IFNγ^+^IL-2^+^ EM cells to the total EM cytokine response towards *Pf*Spz was relatively more pronounced than to that against *Pf*RBC. Specifically, on day C+140 and C+400 the relative contribution of polyfunctional EM cells was 37% [25–62] and 19.2% [16–30] in response to *Pf*Spz, compared to 3.3% [1.5–4.3] and 3.4% [2.0–5.3] in response to *Pf*RBC (p<0.001 and p<0.05 respectively; two-way ANOVA with Bonferroni post-test; data not shown and **Figure 6**). Thus, although infrequent in total number, polyfunctional EM cells with specificity for both *Pf*RBC and *Pf*Spz were readily induced upon exposure, and formed a greater relative contribution to *Pf*Spz than to *Pf*RBC responses.

## Discussion

In this study we delineate the dynamics and composition of cellular immune responses to both sporozoites and asexual blood-stage *Plasmodium falciparum* parasites following infection in previously-naïve individuals. We demonstrate unequivocally that specific IFNγ responses to both stages of the malaria parasite are not only readily induced following infection, but also persist more or less undiminished over at least 14 months in the absence of further exposure. The main contributors to these whole parasite-specific IFNγ responses are γδT cells and CD4^+^ EM T cells, with NK cells making up a smaller remaining fraction of responding cells. We show that not only adaptive, but also semi-innate and innate lymphocytes responses exhibit an immunological re-call pattern and present evidence suggesting that immunological memory for *Pf*RBC is carried within both the αβT cell and γδT cell compartments.

Our demonstration of lengthy persistence of cellular immunological responses following *P. falciparum* infection in humans stands in contrast to the popularly held perception that clinical immunity to malaria is short-lived. As discussed previously by Struik et al. [6] studies reporting such short-lived immunity are mainly anecdotal and few consistent data pro or contra this hypothesis have been published. Our current findings prove that long-lived cellular responses can be adequately maintained, at least when induced under experimental conditions.

A central mediator of such cellular immunological responses to the malaria parasite is the cytokine IFNγ (reviewed in [19]). *In vitro* parasite-specific IFNγ responses have been shown by us and others to associate with protection against malaria both amongst volunteers undergoing experimentally induced infection [21], [22], [23] and naturally-exposed human populations [24], [25], [26], [27]. Phenotypic characterization of the *in vitro* IFNγ response to whole blood-stage parasites (*Pf*RBC) in malaria-naive donors has variously implicated ‘innate’ natural killer (NK) cells [28], [29], [30], ‘semi-innate’ γδT cells [31], [32] (including NK-like γδT cells [33]) and ‘adaptive’ αβT cells [32], [34], [35]. It remains unknown, however, how these intrinsic responses develop and mature in the course of a malaria infection and how (long) cellular memory is maintained.

Consistent with findings by others [31], [33], we show that ‘semi-innate’ γδT cells comprise the largest population of lymphocytes responding with IFNγ production to *Pf*RBC in malaria-naïve donors. Interestingly, this remains largely true following exposure, despite the obvious increase in ‘adaptive’ responses. Two factors may contribute to the overall increase in responding γδT cell numbers: i) The overall proportion of γδT cells in the PBMC pool increases following exposure to parasites, which persists for at least a year. A transient dip in circulating γδT cells during infection, followed by reactive increase afterwards, has been observed before in primary [36], [37] but not in repeated malaria infections [38]. ii) A slightly increased proportion of these γδT cells responds to *Pf*RBC following infection. This increase may represent the recruitment of *Pf*RBC-specific γδT clones to the peripheral circulation or a non-specific bystander effect, since γδT cells can readily respond to *P. falciparum* lysate by proliferation in a polyclonal fashion [39], [40]. Whatever the underlying mechanism, our data suggest that the γδT compartment does contribute autonomously to cellular immunological memory up to 14 months post infection, independently of other PBMC including αβT cells. In contrast, we and others have recently shown that the ‘re-call-like’ response observed in NK cells post infection is in fact fully dependent on αβT cells [30], [41]. These data can be combined into a model in which ‘semi-innate’ γδT cells, ‘adaptive’ αβT cells and ‘innate’ NK cells all contribute to a robust and long-lived IFNγ response following infection with *P. falciparum*, although through different mechanisms. For γδT cells this is largely through an overall expansion of this compartment in peripheral blood, in addition to a minor increase in the proportion of responding γδT cells. For αβT cells the proportional increase in response is also relatively small, but in absolute terms these lymphocytes already make up the vast majority of PBMC populations. NK cells finally, although fewer in absolute terms, show a much larger proportional increase in response, albeit dependent on the increase in T cell responses [30].

The majority of responding T cells displays an EM (CD45RO^+^ CD62L^-^) phenotype, even over a year post infection, at least in donors with sufficient numbers of responding cells to assess this. Whether such a composition is also representative of extremely low responders, or whether those donors exhibit a relative response deficit in a particular lymphocyte sub-set, cannot as yet be determined.

The apparent scarcity of responding CD62L^+^ CM cells may be partly due to the fact that CM cells by definition form only a minor population within the peripheral blood, residing primarily in ‘target’ tissues (e.g., skin and liver) and lymph nodes. Another possibility is that this pattern is inherent to short-term *in vitro* assays such as ours where within the short timeframe of 24 hours, effector memory cells, which are defined by their ability to perform immediate (cytokine producing) effector function, will preferentially respond. Finally, the low number of CD62L expressing responding lymphocytes could be due to loss of CD62L expression, since following antigenic stimulation CM cells can differentiate into an effector memory phenotype and subsequently acquire effector function [42], [43]. Thus the formal compartmental origin of responding cells cannot be determined with certainty from this assay.

The importance of polyfunctional lymphocytes in immunological protection is believed to depend on i) their higher cytokine production [23] and hence more potent effector capability compared to monofunctional cells [44] and ii) their role in the induction and persistence of T cell memory [45]. We recently showed that the development of protection against infection with *P. falciparum* in human volunteers is associated with the induction of IFNγ^+^IL-2^+^ double-positive (polyfunctional) EM T cells in response to *Pf*RBC [22], [23]. Despite an overall increase in the number of responding lymphocytes up to one year post infection, we show here that the relative contribution of polyfunctional cells to the total response remains roughly constant. This may indicate that little differentiation takes place in the functionality of cellular immune responses to *Pf*RBC following exposure [46]. It will be of obvious interest to explore this further in future studies and to determine whether such responses genuinely afford protection.

In contrast to responses to the asexual stage of the malaria parasite, sporozoite-specific cytokine responses have received little attention to date. We find that similar to *Pf*RBC responses, IFNγ responses to *Pf*Spz are readily induced and persist following exposure to infected mosquito bites. Furthermore, as for *Pf*RBC responses, IFNγ production dominates the total cytokine response. A striking feature of the anti-*Pf*Spz response, however, is that polyfunctional IFNγ^+^/IL-2^+^ cells form a relatively larger component of this compared to *Pf*RBC. Whether this represents a genuine acquisition of effector function of the anti-*Pf*Spz response or conversely a failure of these lymphocytes to terminally differentiate into IFNγ single producers [46] remains to be determined.

Our data demonstrate that there is no intrinsic deficit in either the induction or persistence of cellular responses to *P. falciparum* after experimental infection. This raises the obvious question as to why clinical immunity to malaria develops so slowly amongst naturally exposed populations [2], [4]. More specifically, why do cellular responses to *P. falciparum* antigens in naturally exposed donors appear to be so transient/unstable [8], [10], [11], [12], [13], [14], [15] and tend in fact to be *lower* than in non-exposed donors [47], [48]? Several lines of reasoning may help to explain this paradox.

Firstly, by the time treatment is sought by and initiated in patients in resource-poor endemic settings, their parasitemia is typically higher compared to that in our strictly-observed volunteers. High parasitemia has been shown to inhibit the development of immunity both in mice [49] and in humans [11]. This may be due to active suppression or elimination of responding T cells [50], [51] by *P. falciparum*, resulting in reduced *Pf*-specific cellular responses following repeated or chronic infection [11], [47], [48], [52]. Obvious accomplices are regulatory T cells [53], [54], [55], [56], and a comparison of the dynamics of regulatory T cells in natural and experimental infections would be informative in this regard. Secondly, underlying differences in the status of the immune system of inhabitants of the rural tropics may predispose to tolerant, as opposed to sterilizing, immune responses [57]. This may be due to e.g. malnutrition [58] or helminth co-infections [59], [60]. Another factor may be the inherent immaturity in the immune systems of infants and young children, the stage in life at which malaria infections are typically first experienced in endemic settings [61], [62], as well as prior *in utero* exposure [63]. Indeed, IFNγ responses to *P. falciparum* antigens in children tend to be weaker than in adults [14], [64], [65], [66], [67], although of course the effect of prior exposure in these studies cannot be distinguished from that of age per sé. In addition, immunization and *in vitro* PBMC re-stimulation in our experimental infection model were performed with homologous strain parasites, whereas in field studies prior strain exposure varies. Well-described target antigens for protective immunity exhibit high rates of genetic variation, hindering cross-protective immunity in the field [68]. Finally, the immune modulating effects of chloroquine might have enhanced the development of immune responses during the immunization process [69], possibly contributing to the persisting immune responses in Group A.

Despite these caveats in extrapolating our findings to the situation in endemic areas, we show that robust long-lasting cellular immune responses to malaria parasites can be readily induced under experimental conditions, and extend our understanding of how cellular immunological memory to *P. falciparum* develops and is maintained following exposure.

## Materials and Methods

### Parasites

NF54 strain *P. falciparum* asexual blood-stage parasites, regularly screened for mycoplasma contamination, were grown in RPMI-1640 medium containing 10% human A^+^ serum at 5% hematocrit in a semi-automated suspension culture system, in the absence of antibiotics and in an atmosphere containing 3% CO_2_ and 4% O_2_. For *in vitro* stimulation experiments, asynchronous asexual-stage cultures of NF54 strain parasites were harvested at a parasitemia of approximately 5–10% and mature asexual stages purified by centrifugation on a 27% and 63% Percoll density gradient [70]. This purification step results in preparations of 80-90% parasitemia, consisting of more than 95% schizonts/mature trophozoites. Preparations of parasitized red blood cells (*Pf*RBC) were washed twice in PBS and cryopreserved at 150x10^6^/ml in 15% glycerol/PBS in aliquots for use in individual stimulation assays. Cryopreserved *Pf*RBC form almost as strong a stimulus as freshly-prepared *Pf*RBC and have identical stimulatory characteristics (**Figure S6**). Their use in large experiments has logistical advantages, in addition to reducing confounding influences due to inter-batch variation. Mock-cultured uninfected erythrocytes (uRBC) were obtained similarly and served as controls.

Sporozoites were obtained from *Anopheles stephensi* mosquitoes that were reared according to standard procedures in our insectary. Infected mosquitoes were obtained by feeding on gametocyte-containing cultures of NF54 strain *P. falciparum*, as described previously [71]. On day 21–28 after infection, the salivary glands of the mosquitoes were collected by hand-dissection. Salivary glands were collected in RPMI-1640 medium (Gibco) and homogenized in a custom glass grinder. Sporozoites were counted in a Bürker-Türk counting chamber using phase-contrast microscopy. Sporozoites were cryopreserved at 16×10^6^/ml in 15% glycerol/PBS in aliquots for use in individual stimulation assays. Sporozoites that had undergone one freeze-thaw cycle were determined microscopically to be still intact, but were no longer able to glide (assay described in [72]). To control for a possible immune-stimulatory effect of salivary gland remnants in the sporozoite preparation, salivary glands from an equal number of uninfected mosquitoes (MSG) were obtained similarly and served as a background control.

### Human ethics statement

All volunteers were recruited after giving written informed consent. The study was approved by the Institutional Review Board of the Radboud University Nijmegen Medical Centre (CMO 2006/207).

### Human infections

The basic design and outcome of experimental human malaria infections at our centre has been described before [73]. For the study presented here [23], 15 healthy malaria naïve Dutch volunteers were recruited and randomized double-blind to either Group A (n = 10) or Group B (n = 5). Group A volunteers were immunized by exposure on three occasions, at monthly intervals, to the bites of 12-15 NF54 strain *P. falciparum*-infected mosquitoes, whilst continuously taking a standard prophylactic regimen of chloroquine (300mg base per week). Group B volunteers similarly took chloroquine and were exposed to the same number of bites, but from uninfected mosquitoes. Two months after the final exposure and one month after discontinuation of chloroquine prophylaxis, all 15 volunteers were challenged by exposure to the bites of 5 *P. falciparum*-infected mosquitoes and followed-up closely for symptoms and signs of malaria. As soon as they were found to be thick blood-smear positive, volunteers were treated with a standard curative regimen of artemether/lumefantrine (AL) consisting of six doses of 13 80/480 mg over three days. Duration and peak height of parasitemia in volunteers following each round of infection, as measured retrospectively by PCR [23], is shown in **Table S2**.

### Cellular immunology

Venous whole blood was collected into citrated CPT vacutainers (Becton and Dickinson, Basel) at inclusion (day I-1), and immediately prior to challenge (day C-1), during expected blood-stage malaria infection (day C+9), two weeks after treatment with AL (day C+35) and again 4.5 months (day C+140) and 1.1 year (day C+400) after challenge (**Figure 1**). Peripheral Blood Mononuclear Cells (PBMC) were obtained by density gradient centrifugation, washed three times in cold PBS, enumerated, frozen down in fetal-calf serum containing 10% dimethylsulfoxide and stored in liquid nitrogen. Immediately prior to use, cells were thawed, washed twice in RPMI and re-suspended in complete culture medium (RPMI 1640 Dutch modification (Gibco) containing 2 mM glutamine, 1mM pyruvate, 50 µg/ml gentamycine and 10% human A^+^ serum, (Sanquin, Nijmegen)) for a final concentration of 2.5×10^6^/ml. PBMC were transferred into 96-well round-bottom plates and stimulated in duplicate wells with either 5x10^6^/ml (final concentration) cryopreserved *Pf*RBC or uRBC, or 5.6×10^5^/ml cryopreserved sporozoites or the extract of an equivalent number of uninfected mosquito salivary glands in a total volume of 200 µl/well for 24 hours at 37°C/5%CO_2_. Dose and duration of stimulation were chosen based on earlier optimization assays. Initial experiments included samples from time points I-1 through C+35; in a later set of experiments, time points C+140 and C+400 were compared. In a subset of experiments, PBMC from time points I-1 through C+35 were instead stimulated with protein pools of individual purified sprorozoite-stage (CSP and TRAP), liver-stage (LSA-1 or Exp-1) or blood-stage (AMA-1, MSP-2, MSP-3 and GLURP) antigens in concentrations of both 5 and 30 µg/ml per antigen. Full length CSP [74] was kindly provided by A. Birkett, TRAP MR149A, MSP-2 MR141 [75], *Pf*Exp-1 MR95 [76] by G. Corradin, MSP-3 [77] by C. Oeuvray, GLURP [78] by M. Theisen, LSA-1 [79] by T. Richie and AMA-1 [80] by A. Thomas. In these latter experiments 60 IU human recombinant IL-2 (Proleukin, Novartis) was added to the culture medium for optimal cellular responses. In all experiments, 100μL/well supernatant was removed 4 hours prior to cell harvest and replaced with 100μL/well fresh culture medium containing Brefeldin A (Sigma) with a final concentration of 10μg/ml.

### Depletion/recombination

For recombination experiments, PBMC collected at inclusion (I) and post-challenge (C) from seven donors from Group A and B for whom sufficient cells were available, were divided into two aliquots. For two of these donors, cells from day 35 post-challenge were used and for the other five donors C+140 cells. One aliquot of each sample was depleted of γδT cells by magnetic beads, whereas untouched γδT^+^ cells were isolated from the second aliquot by negative selection (Anti-TCR γ/δ MicroBead Kit and TCRγ/δ^+^T Cell Isolation Kit, respectively, both from Miltenyi Biotech), according to the manufacturer’s instructions. Following separation, autologous I/C γδT^-^ and γδT^+^ cells were recombined at their original ratios. Purity of depletion was consistently >90%, whereas purity of negative selected untouched γδT^+^ cells varied between 40–80%. The majority of contaminating non-γδT cells in these negatively selected populations consisted of NK and other non-T lymphocytes. Since the proportion of γδT^+^ cells added directly reflected the proportion of these cells in the PBMC population (I or C) from which they were derived (see also **Table S1**), this proportion was higher in wells containing C γδT^+^ cells than in wells containing I γδT^+^ cells : 1.5 [1.1–2.1], 4.7 [2.6–9.1], 1.4 [1.1–1.8] and 3.9 [2.8–4.0] (% of lymphocytes [IQR]) respectively for I+I, I+C, C+I and C+C.

### Intracellular staining for flow cytometry

CD3-CD56-γδT stain (all time points): Following 24 hour of *in vitro* stimulation, PBMC were harvested and transferred to FACS tubes (250,000 cells/tube), washed once in FACS buffer (0.5% BSA/PBS) and incubated for 15 minutes in 100 µl FACS buffer with fluorochrome-labelled mAbs against the cell-surface markers CD3-PerCP (clone CK7, BD Biosciences), TCR Pan γ/δ-PE (clone IMMU510, Beckman Coulter, Fullerton, CA, USA) and CD56-APC (clone MEM188, eBioscience San Diego, CA, USA). Cells were washed again in FACS buffer and incubated for 15 minutes in 100 µl fixation medium A (Caltag Laboratories, Carlsbad CA) according to the manufacturer's instructions, washed and incubated for 15 minutes with IFNγ-FITC (clone 4S.B3, eBioscience) in 100 µl permeabilization medium B. After a final wash step, cells were re-suspended in FACS buffer and acquired on a FACScalibur flow cytometer (Becton Dickenson). **Figure S1.A** shows the gating strategy for this staining.

Effector memory phenotyping stain (I-1, C-1, C+9, C+35): Following the procedure described above, cells were stained with CD45RO-PE (clone UCHL1), CD62L-PE-Cy7 (clone DREG56), IFNγ-FITC (clone 4S.B3) and IL-2-APC (clone MQ1-17H12, all eBioscience). **Figure S1.B** shows the gating strategy for this staining.

Additional T cell phenotyping stain for C+140 and C+400: Following 24 hour of *in vitro* stimulation, PBMC were harvested and transferred to 96 wells V-plate (500,000 cells/well), washed once in PBS and incubated with 50 µl Live/Dead fixable dead cell stain kit Aqua (Invitrogen, Carlsbad, CA, USA) in PBS for 30 min on 4°C. Cells were washed in PBS and for a second time with FACS buffer (PBS containing 0.5% albumin for bovine serum (Sigma Chemical Co.)), and stained in 50 µl FACS buffer with anti-TCR Pan γ/δ-PE (clone IMMU510, Beckman-Coulter), CD45RO-ECD (clone UCHL1, Beckman-Coulter), CD3-PerCP (Clone UCHT1, BioLegend, San Diego, CA, USA), CD62L-PE-Cy7 (Clone DREG56, eBioscience), CD4-Pacific Blue (Clone OKT4, eBioscience) and CD8a-Alexa-fluor 700 (clone HIT8a, BioLegend) for 20 min at 4°C. After washing, cells were incubated with 50 µl fixation Medium A (Caltag, S. San Francisco, CA, USA) and subsequently, incubated with anti-IFNγ-FITC (clone 4S.B3, eBioscience) and IL-2-APC (Clone MQ1-17H12, eBioscience) in 50 µl permeabilization Medium B (Caltag) for 20 min at 4°C. Lymphocytes (100,000) gated by forward- and side-scatter characteristics were acquired on a CyAn ADP 9-color flow cytometer (Beckman- Coulter). **Figure S1.C** and **S1.D** show the gating strategy for this staining.

### Flow cytometry analysis

Flow cytometry analysis was performed using Cell Quest and FlowJo V9.1 software. Gating of lymphocytes and subsequent subgroups was performed as shown in **Figure S1**. Gating of cells positive for IFNγ and/or IL2 was performed using a cut-off based on the geometric mean of cells cultured in medium only.

### Statistical analysis

Statistical analysis were performed using GraphPad Prism 5. Differences in responses within volunteers between multiple time points or between stimuli were analyzed by repeated measures one-way ANOVA with Dunnett's or Bonferroni post-hoc test, as appropriate. Paired/repeated measures analysis was carried out exclusively on complete data sets obtained within a single experiment. Two-way analysis with Bonferroni post-test was performed in order to analyze data sets with multiple variables (both time points and stimuli). One donor had to be excluded from all statistical analysis due to an extreme, but highly variable, outlying IFNγ response to *Pf*RBC at time point C+35. All statistical analyses were performed on data corrected for background: background responses were subtracted from the responses to parasite stimuli for every volunteer at every time point individually (*Pf*RBC - uRBC; sporozoite – mosquito salivary gland; parasite antigens – medium only); negative values were set to zero. P-values <0.05 were considered statistically significant in all analyses.

## Supporting Information

Figure S1 **Representative flow cytometry plots.** **A**. CD3-CD56-γδT stain (all time points). Following 24-hour *in vitro* stimulation, live PBMC were gated based on CD3 expression and sub-populations subsequently further gated based on γδT and CD56 expression. **B.** Effector memory phenotyping stain (I-1, C-1, C+9, C+35). Following 24-hour *in vitro* stimulation, lymphocytes were gated based on their forward-sideward scatter and further gated based on CD45RO/CD62L expression. **C.** Additional T cell phenotyping stain for C+140 and C+400. Following 24-hour *in vitro* stimulation, only viable single cells were gated. Lymphocytes were gated based on their forward-sideward scatter and further sub-gated by CD3 and subsequently CD8/CD4 expression or γδ-TCR expression. To allow comparison with staining B, CD45RO/CD62L cells were assessed without preceding CD3 gating. **D.** Intracellular IFNγ expression of T-cells, CD4 T-cells and CD8 T cells following incubation with *Pf*RBC (column 1), uRBC (column 2), *Pf*Spz (column 3) and MSG (column 4) in lymphocytes obtained from a volunteer post-challenge.
(TIF)

Figure S2 **IFNγ responses by different lymphocyte subsets following** ***in vitro*** **stimulation with** ***Pf*** **RBC.** PBMC were isolated from volunteers prior to immunization (day I-1), immediately prior to patent challenge (day C-1), during expected blood-stage malaria infection (day C+9), two weeks after treatment (day C+35), 4.5 months post-challenge (day C+140) and again 1.1 year post-challenge (day C+400). Cells were stimulated *in vitro* for 24 hours with *Pf*RBC or controls, then stained for phenotype and intracellular IFNγ and analyzed by flow cytometry (gating strategy illustrated in **Figure S1**). Shown are the percentage of αβT cells (**A** - CD3^+^γδ^-^CD56^-^), γδT cells (**B** - CD3^+^γδ^+^CD56^-^), NK cells (**C** - CD3^-^γδ^-^CD56^+^), αβNKT cells (**D** - CD3^+^γδ^-^CD56^+^) and ‘γδNKT’ cells (**E** - CD3^+^γδ^+^CD56^+^) staining positive for IFNγ at each time point for volunteers of Group A (left panels) and Group B (right panels). Background responses were subtracted from the responses to *Pf*RBC for every individual volunteer at every individual time point. Symbols represents responses by individual Group A volunteers (left, n = 10) and Group B volunteers (right, n = 5) for whom sufficient cells were available. Horizontal lines represent group medians.
(TIF)

Figure S3 **Contribution of CD4** ^+^ **and CD8** ^+^ **cells to the total T lymphocyte IFNγ response to** ***Pf*** **RBC and** ***Pf*** **Spz.** PBMC isolated from volunteers at C+400 were stimulated *in vitro* for 24 hours with *Pf*RBC or *Pf*Spz and stained for CD3, CD4 and CD8 (see **Figure S1C**). Data represent median values from Group A and Group B volunteers. Donors with insufficient numbers of IFNγ responding cells to assess the relative contribution of cell subsets were excluded from composition analysis. Numbers of donors included are indicated in each box.
(TIF)

Figure S4 **Total IL-2 and polyfunctional responses by EM cells to** ***Pf*** **RBC and** ***Pf*** **Spz.** PBMC of volunteers of Group A and Group B were stimulated *in vitro* for 24 hours with *Pf*RBC or *Pf*Spz or their respective uRBC or MSG controls, then stained for intracellular IFNγ and IL-2 and analyzed by flow cytometry. Shown are the percentages of effector memory (EM) cells staining positive for IL-2 **(A)** or both IFNγ and IL-2 **(B)** at each time point. Gating strategies are shown in **Figure S1**. Background responses were subtracted from the responses to parasite stimuli for every individual volunteer at every individual time point. Symbols represents responses by individual Group A volunteers and Group B volunteers for whom sufficient cells were available. Horizontal lines represent group medians and IQR.
(TIF)

Figure S5 **Contribution of polyfunctional EM cells to total IFNγ and/or IL-2 producing EM cells.** PBMC of volunteers of Group A (**A+C**) and Group B (**B+D**) were stimulated *in vitro* for 24 hours with *Pf*RBC (**A+B**) or *Pf*Spz (**C+D**) or their respective uRBC or MSG controls, then stained for intracellular IFNγ and IL-2 and analyzed by flow cytometry. Shown are percentage contributions of polyfunctional (IFNγ and IL-2) cells to the total number of IFNγ and/or IL-2 producing effector memory (EM) cells at each time point, after correction for background for every individual donor. Gating strategies are shown in Figure S1. Symbols represents individual Group A volunteers (n = 10) and Group B volunteers (n = 5) for whom sufficient cells were available. Horizontal lines represent group medians and IQR.
(TIF)

Figure S6 **IFNγ responses by PBMC following** ***in vitro*** **stimulation with cryopreserved or fresh** ***Pf*** **RBC. A-F.** PBMC were stimulated for 20 hours *in vitro* in the presence of 5x10^6^/ml either freshly isolated or cryopreserved *Pf*RBC from the same batch, equivalent numbers of uRBC, 10 µg/ml PHA or RPMI only. The collected supernatant was stored at -80°C until subsequent cytokine measurement. IL-1β (**A**), IL-2 (**B**), IL-10 (**C**), IL-12p70 (**D**), IFNγ (**E**) and TNFα (**F**) secretion into the supernatant was detected by commercially available microbead array according to the manufacturer's instructions (Bio-Rad, Veenendaal, The Netherlands). Data represent mean+SEM for five donors.
(TIF)

Table S1 **Lymphocyte subset composition in volunteers prior to, during and post infection.**
(RTF)

Table S2 **Parasitemia in volunteers following immunizations and challenge, determined by PCR.**
(RTF)
