# Supplementary figures and images for: Longevity and Composition of Cellular Immune Responses Following Experimental Plasmodium falciparum Malaria Infection in Humans
Source: PLoS Pathog. 2011 Dec 1;7(12):e1002389. doi: 10.1371/journal.ppat.1002389 (PMC3228790; doi:10.1371/journal.ppat.1002389)

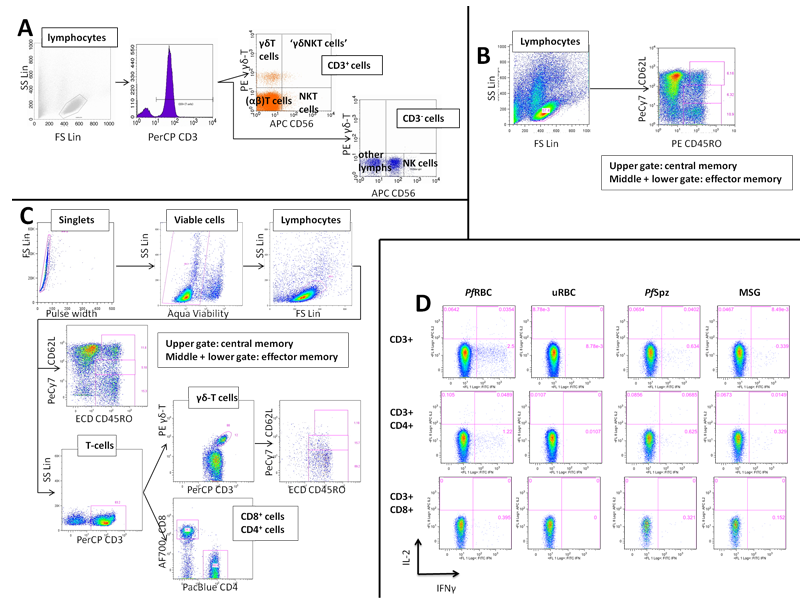

Supplement: Figure S1 — Representative flow cytometry plots. A. CD3-CD56-γδT stain (all time points). Following 24-hour in vitro stimulation, live PBMC were gated based on CD3 expression and sub-populations subsequently further gated based on γδT and CD56 expression. B. Effector memory phenotyping stain (I-1, C-1, C+9, C+35). Following 24-hour in vitro stimulation, lymphocytes were gated based on their forward-sideward scatter and further gated based on CD45RO/CD62L expression. C. Additional T cell phenotyping stain for C+140 and C+400. Following 24-hour in vitro stimulation, only viable single cells were gated. Lymphocytes were gated based on their forward-sideward scatter and further sub-gated by CD3 and subsequently CD8/CD4 expression or γδ-TCR expression. To allow comparison with staining B, CD45RO/CD62L cells were assessed without preceding CD3 gating. D. Intracellular IFNγ expression of T-cells, CD4 T-cells and CD8 T cells following incubation with PfRBC (column 1), uRBC (column 2), PfSpz (column 3) and MSG (column 4) in lymphocytes obtained from a volunteer post-challenge. (TIF) [file ppat.1002389.s001.tif]

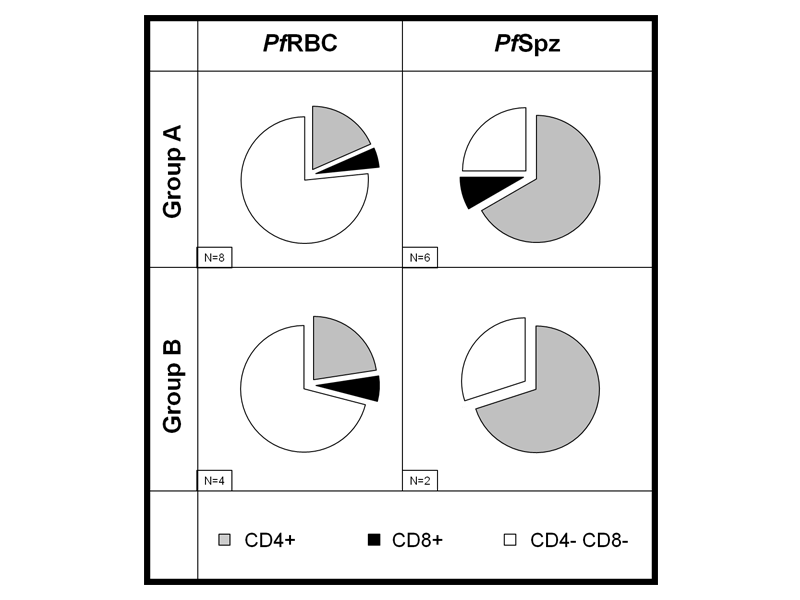

Supplement: Figure S3 — Contribution of CD4 + and CD8 + cells to the total T lymphocyte IFNγ response to Pf RBC and Pf Spz. PBMC isolated from volunteers at C+400 were stimulated in vitro for 24 hours with PfRBC or PfSpz and stained for CD3, CD4 and CD8 (see Figure S1C). Data represent median values from Group A and Group B volunteers. Donors with insufficient numbers of IFNγ responding cells to assess the relative contribution of cell subsets were excluded from composition analysis. Numbers of donors included are indicated in each box. (TIF) [file ppat.1002389.s003.tif]
