# Supplementary material for: Longevity and Composition of Cellular Immune Responses Following Experimental Plasmodium falciparum Malaria Infection in Humans
Source: PLoS Pathog. 2011 Dec 1;7(12):e1002389. doi: 10.1371/journal.ppat.1002389 (PMC3228790; doi:10.1371/journal.ppat.1002389)
Supplement: Table S1 — Lymphocyte subset composition in volunteers prior to, during and post infection. (RTF) [file ppat.1002389.s007.rtf]

Supplementary Table 1:  Lymphocyte subset composition in volunteers prior to, during and post infection.

	Time point	ãäT cells	ãäNKT cells	(áâ)T cells	(áâ)NKT cells	NK cells	other 
lymphocytes	

Group A	
I-1	
2.4
(1.6-3.3)	
0.4
(0.2-0.5)	
77.4
(76.4-80.3)	
0.8
(0.6-1.2)	
4.6
(3.5-7.1)	
14.1
(11.6-15.3)	
(n=10)	C-1	3.0
(2.3-4.3)	0.3
(0.2-0.5)	77.4
(76.6-80.4)	0.7
(0.4-1.1)	2.7
(1.9-5)	13.3
(12.9-16)	
	C+9	3.5
(3-4.1)	0.3
(0.3-0.8)	77.4
(75.2-79)	0.7
(0.4-1)	2.8
(2.5-5.4)	14.4
(11.9-16.2)	
	C+35	4.3
(3.8-5.5)	0.6
(0.4-0.8)	75.2
(72.9-79)	0.8
(0.6-1.2)	4.9
(2.4-7.9)	12.2
(11.6-14.6)	
	C+140	4.3
(2.7-6.2)	0.6
(0.5-1.1)	73.7
(69.1-76.2)	1.0
(0.8-2.6)	3.6
(2.8-7.2)	13.2
(12.7-15.9)	
	C+400	3.7
(3.6-4.5)	1.2
(0.9-1.6)	74.4
(71.8-75.5)	1.2
(0.8-2.1)	6.4
(5.2-9.8)	11.4
(9.9-13.2)	

Group B	
I-1	
1.2
(0.8-1.4)	
0.1
(0.1-0.2)	
80.9
(80-82.1)	
0.6
(0.6-0.8)	
2.6
(2.6-2.9)	
13.4
(10.6-14.4)	
(n=5)	C-1	0.8
(0.6-1.3)	0.1
(0-0.2)	78.0
(77.3-81.1)	0.4
(0.3-0.6)	3.2
(2.3-4)	17.3
(10.5-17.5)	
	C+9	0.7
(0.6-0.9)	0.1
(0.1-0.2)	79.0
(75.1-83.8)	0.6
(0.3-0.7)	3.1
(2.4-4)	16.4
(11.7-17.3)	
	C+35	2.2
(1.9-3)	0.3
(0.3-0.4)	79.8
(76-82.5)	0.8
(0.5-2.8)	4.0
(3.7-4.3)	12.2
(9.8-14.2)	
	C+140	2.8
(2.8-3.5)	0.5
(0.5-0.6)	77.1
(75.5-79.2)	0.8
(0.7-0.8)	4.6
(3.6-6.8)	12.8
(10.8-14.5)	
	C+400	2.3
(2-2.6)	0.5
(0.5-0.8)	77.0
(76.1-78.7)	1.0
(0.8-1.3)	5.5
(4.5-6.7)	12.7
(10.8-14.1)	

All data represent median (IQR) percentage of total lymphocytes.
