# Supplementary material for: Longevity and Composition of Cellular Immune Responses Following Experimental Plasmodium falciparum Malaria Infection in Humans
Source: PLoS Pathog. 2011 Dec 1;7(12):e1002389. doi: 10.1371/journal.ppat.1002389 (PMC3228790; doi:10.1371/journal.ppat.1002389)
Supplement: Table S2 — Parasitemia in volunteers following immunizations and challenge, determined by PCR. (RTF) [file ppat.1002389.s008.rtf]

Supplementary Table 2: Parasitemia in volunteers following immunizations and challenge, determined by PCR.


Group A	No. PCR+/total volunteers	Median peak parasite density/ml whole blood [range] 1
	Median duration PCR+  in days [range]1 	

Immunization 1	
10/10	
16171 [55-137454]
	
2 [1-4]	
Immunization 2	6/10	1567 [467-3360]
	1.5 [1-2]	
Immunization 3	3/10	1262 [45-1436]
	1 [1-2]	

Challenge	
0/10	
n/a	
n/a	


Group B	No. PCR+/total volunteers	Median peak parasite density/ml whole blood [range] 1
	Median duration PCR+  in days [range]1	

Immunization 1	
0/5
	
n/a	
n/a	
Immunization 2	0/5
	n/a	n/a	
Immunization 3	0/5
	n/a	n/a	

Challenge	
5/5	
16937 [4491-34543]
	
4 [2-5]	

1 for PCR+ volunteers only
n/a = not applicable
